# Parent Representatives' Experiences of Participating in Children's Disability Network Team Service Improvement Groups in Ireland: A Qualitative Study

**DOI:** 10.1111/hex.70962

**Published:** 2026-11-05

**Authors:** Geraldine Moran, Noreen O'Leary, Katie Robinson, Hannah Christie, Alison Grant, Alyssa Garland, Eileen Doyle, Rosa Tomro‐Hofmann, Aoife Lily Gallagher

**Affiliations:** ^1^ School of Allied Health University of Limerick Faculty of Education and Health Sciences Limerick County Limerick Ireland; ^2^ Health Research Institute University of Limerick Limerick Ireland; ^3^ Health Professions Education Centre Royal College of Surgeons in Ireland Dublin 2 Ireland; ^4^ School of Population Health Royal College of Surgeons in Ireland Dublin 2 Ireland; ^5^ PPIE Parent Advisory Group Ireland

**Keywords:** Children's Disability Network Teams (CDNTs), neurodevelopmental disability, parent representatives, patient and public involvement and engagement (PPIE), qualitative research, rehabilitation services, service improvement, thematic analysis

## Abstract

**Background:**

Effective patient and public involvement and engagement (PPIE) is an essential element of a quality healthcare system. In recent years, public health services for children with disabilities in Ireland have sought to include the voice of parent representatives in service improvement. Such initiatives provide important learning opportunities for comparable public health systems internationally.

**Objective:**

This study aimed to explore parents' experiences of the representative role within Ireland's Children's Disability Network Teams. Understanding these experiences can inform the development and implementation of meaningful PPIE in service development in Ireland and beyond.

**Design:**

A descriptive qualitative study was undertaken with guidance from a PPIE parent advisory group. Data were gathered via semi‐structured online interviews, transcribed and analysed thematically. Findings were reviewed by the PPIE parent advisory group.

**Setting and Participants:**

Eleven participants were recruited to the study from a diverse range of health regions across Ireland. All participants had direct experience as a member of a parent representative forum related to children's disability services.

**Results:**

Four themes were identified: (i) From the individual to the collective, parent motivations to become a representative; (ii) An ambiguous and informal role, with unintended consequences; (iii) The health system as a controlling and transactional organisation; and (iv) Unheard, unseen, and unsafe: The burden of representation.

**Conclusions:**

Participants described experiences which reveal psychological, logistical and organisational barriers that limited the possibility of meaningful partnership and influence within service improvement processes. The findings demonstrate the importance of ensuring that the role of parent representative is clearly defined and formalised, and of the need to provide support and training around the role for parents. The study findings also highlight the importance of ensuring parents are properly recompensed for their time and efforts. Enabling parent representatives to connect informally with one another may mitigate the emotional burden of the role. More broadly, the findings suggest that meaningful partnership requires not only formal involvement structures but also transparency, trust and genuine opportunities to influence decision‐making.

**Lived Experience:**

PPIE contributors collaborated in the study design and developed the interview guide, supported recruitment, contributed to interpretation and refinement of themes, informed the discussion of findings, and reviewed the final manuscript.

## 1 Introduction

Globally, patient and public involvement and engagement (PPIE) is widely promoted as a cornerstone of quality improvement and person‐centred care, embedding service‐user perspectives in the design, delivery and governance of health services [1, 2]. Many national health and social care policies now position PPIE as an integral component of safe, effective and equitable healthcare, rather than as a discretionary add‐on [2, 3]. PPIE is an umbrella term encompassing different approaches to involving patients, service users, families and the public in healthcare, including consultation, engagement, co‐design, co‐production and shared decision‐making [4, 5]. Despite this policy momentum, the literature cautions that PPIE remains vulnerable to practises that confer little genuine influence, undermining trust, marginalising diverse voices, and limiting impact. Persistent challenges relating to unequal power and role clarity appear to constrain meaningful involvement, emphasising the need for more equitable approaches to shared decision‐making [4].

A recent mapping review of 37 systematic reviews of PPIE in health service research identified substantial inconsistencies in the reporting of PPIE processes and outcomes, alongside limited evidence on the experiential, psychological and relational aspects of involvement at organisational and governance levels [6]. Participants highlight unclear structures, limited influence and tokenistic practices across the included studies. The review concluded that more robust qualitative research is needed to better understand experiences of involvement and the conditions that support meaningful, person‐centred PPIE.

Empirical studies examining patient, family and service‐user representatives within healthcare working groups, advisory structures and service‐improvement initiatives have highlighted particular complexities associated with representative roles. These studies also identify uncertainty around roles and expectations, questions of representativeness, unequal opportunities to influence decision‐making, and the importance of organisational support and communication in enabling meaningful participation [7, 8, 9, 10]. Collectively, the literature suggests that formal inclusion in healthcare governance and service‐improvement structures does not in itself ensure meaningful representation or influence.

In Ireland, children's disability services are delivered through Children's Disability Network Teams (CDNTs), which provide interdisciplinary services to children and young people with complex needs [11, 12]. Partnership with families is central to the national CDNT governance framework, with the HSE committing to strengthening family engagement and formalising Family Forums and Family Representative Groups within CDNT governance [11, 12, 13]. Family Forums provide opportunities for parents and carers to discuss local disability services, identify priorities for improvement, and elect Family Representatives who engage with CDNT managers and participate in regional and national representative structures to inform service planning and development [11, 12]. Within the governance framework, Family Representatives are intended to convey the perspectives and priorities of families from local Family Forums into local, regional and national service planning and improvement processes. An interim national mixed‐methods study commissioned by the Health Service Executive (HSE), based on survey data (*n* = 202) and multi‐stakeholder focus groups, identified challenges relating to unclear purpose, inconsistent implementation and psychological safety, alongside a need for clearer governance and facilitation [14]. However, no published empirical research has examined parent representatives' experiences of participation in CDNT service improvement groups in Ireland.

The aim of this study was to explore parent representatives' experiences of participating in service improvement groups within Ireland's CDNTs.

## 2 Methods

### 2.1 Study Design and Methodological Orientation

The study is underpinned by a social constructivist ontology, recognising that knowledge and meaning are co‐constructed through social interaction and shaped by contextual, relational and cultural processes [15]. This orientation aligns with the study's aim of understanding how parents interpret and give meaning to their representative roles within a complex service system. This study adopted a qualitative research design using semi‐structured interviews to explore parent representatives' experiences within children's disability services. The COREQ guidelines were used to support transparent and comprehensive reporting of the study procedures and findings [16]. See Appendix S1.

### 2.2 Study Context

In Ireland, publicly funded children's disability services are delivered by the Health Service Executive (HSE) and partner organisations. Historically fragmented service provision prompted reforms under the Progressing Disability Services (PDS) programme, which established a national network of 93 Children's Disability Network Teams (CDNTs) operating through a needs‐based, family‐centred model of interdisciplinary care [11, 12, 17]. CDNTs provide services to children and young people with complex neurodevelopmental disabilities whose needs require support from an interdisciplinary team.

### 2.3 Ethical Considerations

Ethical approval for this study was granted by the University of Limerick Faculty of Education and Health Sciences Research Ethics Committee (REC Ref: 2025_06_06_EHS) prior to commencement of the study. All participants received detailed written and verbal information about the study and provided written informed consent prior to participation. Participation was voluntary, and individuals were informed that they could withdraw from the study at any point up to the stage at which their data were anonymised.

### 2.4 Research Team and Reflexivity

The research team consisted of three speech and language therapy researchers (one PhD student and two PhD‐qualified researchers), one occupational therapist (PhD), one psychologist (PhD), two PPIE parent advisors and two MSc speech and language therapy students. Two female researchers conducted the interviews, and both brought clinical and academic experience in neurodevelopmental disability, with one researcher drawing on 16 years of clinical practice with this population. While one researcher had not received formal qualitative interviewing training, the other had completed such training and provided methodological support throughout the process. Neither interviewer had any prior clinical, educational or personal relationships with the participants. Participants were informed of the researchers' institutional affiliations and the purpose of the research prior to participation.

As a team with a service‐provider background, we recognised the potential influence of our professional assumptions and insider perspectives on data interpretation. Reflexive practices were embedded throughout the study through researcher memos, post‐interview debriefing and team reflexive discussions. Engagement with the PPIE parent advisory group further informed reflexivity by bringing a lived‐experience perspective to the interpretation of emerging findings.

### 2.5 Patient and Public Involvement and Engagement (PPIE)

PPIE was embedded throughout the research process. The PPIE parent advisory group contributed at key stages including study design, collaborative development of the interview guide, recruitment, interpretation of findings, refinement of themes and review of the final manuscript. The group comprised three parents with relevant lived and representative experience. Two members had direct experience of CDNT services, while the third brought lived experience of caring and extensive experience in PPIE and representative forums. The PPIE contribution is reported according to the Guidance for Reporting Involvement of Patients and the Public 2 Short Form (GRIPP2‐SF) checklist, an internationally recognised framework for reporting patient and public involvement in research ([18]; see Appendix S2).

### 2.6 Participant Recruitment and Sampling

A purposive sampling strategy was used to recruit parent representatives with experience of involvement in CDNTs. As no single mechanism existed for identifying and contacting parent representatives across Ireland, recruitment relied on collaboration with national and regional disability organisations, parent‐led advocacy networks, parent support groups and disability NGOs, as well as support from the PPIE parent advisory group. These organisations acted as gatekeepers and were invited to distribute the study flyer within their networks. As recruitment materials were disseminated through multiple organisations and networks, the number of individuals who received the study invitation could not be determined.

The recruitment strategy was informed by the concept of information power [19]. The focused research question and participants' specific experience of representative roles supported sufficient information power to address the study aim. Recruitment across geographical regions and different levels of representation also provided variation in relevant experience within the sample. Consistent with reflexive thematic analysis, sample size was guided by information power rather than a formal assessment of data saturation.

Study materials were circulated via networks as described above. Interested individuals were invited to contact the research team directly to express interest in participation. Eligible participants were then invited to attend an information session prior to providing informed consent and scheduling an interview. Efforts were made to recruit participants from diverse geographical locations and to include both male and female parent representatives. Eleven participants volunteered to participate in the study. No eligible participants declined participation following enrolment, and no participants withdrew from the study. To protect confidentiality within a relatively small and potentially identifiable population of parent representatives, participant characteristics are reported in aggregate form only.

### 2.7 Data Collection Procedures

Data were collected through individual online interviews, conducted between August and October 2025. The interview guide was developed collaboratively with the PPIE parent advisory group, drawing on their experiential knowledge as parents of children with disabilities and their experience and understanding of family engagement in services. It comprised seven core questions exploring experiences in the role of parent representative (see Appendix S3). The interview guide was not formally pilot tested, and the core interview questions remained unchanged throughout data collection. One researcher conducted nine interviews and the second researcher conducted two, both from private offices, while participants joined primarily from their homes. Cameras remained on throughout, and privacy was confirmed verbally at the outset of each interview. No non‐participants were present in the interviewers' locations. Brief interruptions by participants' children occurred on occasion during interviews conducted in participants' homes. Interviews lasted 45–90 min averaging 60 min and were recorded using MS Teams. Repeat interviews were not conducted.

Handwritten contextual field notes were taken during each interview to document environmental context, emotional tone and initial analytic reflections. Participants were offered breaks when required, including in response to child interruptions, emotional distress or connectivity issues; one participant turned off their camera to stabilise reception. Recordings were transcribed using MS Teams' automatic transcription function. Transcripts were reviewed against the original audio/video recordings and amended where necessary to correct transcription errors before analysis commenced. Identifying information was removed during transcript preparation. Transcripts were not returned to participants for comment or correction. The final data set comprised 11 anonymised transcripts for analysis.

### 2.8 Data Analysis

Reflexive thematic analysis was selected as it offers a rigorous and flexible approach suited to examining experience‐based qualitative accounts in disability research [20]. An inductive, analytic process was used to ensure that categories and themes were grounded in participants' accounts rather than shaped by predetermined theoretical assumptions. Analysis followed Braun and Clarke's six‐phase framework [21]. The research team undertook familiarisation through repeated transcript reading, analytic memoing, and the development of preliminary visual maps. The lead author generated initial line‐by‐line codes in NVivo 15 [22]. Codes and developing interpretations were subsequently discussed with a second reviewer and reviewed through collaborative analytic discussions involving the wider research team. Rather than aiming to ‘resolve discrepancies’, the collaborative dialogue supported reflexive engagement with the data and enhanced the trustworthiness of the analytic process [20, 23].

Two members of the research team organised codes into categories and generated early thematic structures. These were refined during two analysis meetings that incorporated visual diagramming, mapping of codes and collective interpretive discussion. The full research team, including the PPIE parent advisory group, contributed to discussions regarding the development, refinement and naming of themes, bringing lived‐experience perspectives to the interpretive process. Trustworthiness of the analysis was supported through reflexive journaling, maintenance of an audit trail, collaborative analytic discussions, peer debriefing, and prolonged engagement with the data set. Across the data set, 233 open codes were generated and organised into 14 categories, which informed the development of four overarching themes. A summary of the analytic progression from open codes to categories and themes is provided in Appendix S4. Participants were provided with the findings to review whether anonymity had been adequately preserved and to offer any comments.

## 3 Results

### 3.1 Participant Backgrounds

Eleven participants were recruited. Nine were women and two were men. Participants were recruited from five of Ireland's six Regional Health Areas (RHAs), providing broad geographical representation and reflecting experiences from both urban and rural service contexts. All participants were parents of children aged 0–18 years with complex neurodevelopmental disabilities. Participants had experience of a range of representative roles across local, regional and national forums related to children's disability services in Ireland. Participants ranged from newly appointed representatives to those with approximately 4–5 years' involvement, collectively encompassing a diverse range of experience over time.

### 3.2 Results of Analysis

Four overarching descriptive themes were identified: (i) From the individual to the collective, parent motivations to become a representative; (ii) An ambiguous and informal role, with unintended consequences; (iii) The health system as a controlling and transactional organisation; and (iv) Unheard, unseen, and unsafe: The burden of representation.

## 4 Theme 1: From the Individual to the Collective, Parent Motivations to Become a Representative

This theme captures how parents' individual experiences of service receipt motivated them to get involved as a representative. All participants described having experienced significant challenges in accessing services for their own child, explaining various inadequacies they had encountered in relation to the current service model. These included long waiting times, limited access to interventions, inconsistent and fragmented service provision, and inadequate follow‐up:

The system isn't working and it is in complete crisis.

(Participant 4)

Prior attempts to advocate for their child through complaints or individual engagement with services were described as ineffective by most participants. Becoming involved as a parent representative was described by all participants as an opportunity to move beyond their individual negative experiences and to influence service improvement for others. This shift from individual to collective advocacy was reflected in one participant's account:

There was zero supports…. I suppose I kind of frankly had enough and I was like, yes, I'm happy to be a voice and I should use it and I'm well able to articulate myself and so when the opportunity came up to be on the Family Forum, I put myself forward.

(Participant 10)

## 5 Theme 2: An Ambiguous and Informal Role, With Unintended Consequences

The representative role was described by many participants as poorly defined, ineffective and lacking clear mechanisms and processes necessary to support meaningful participation. This lack of clarity led some participants to interpret the role for themselves and to undertake advocacy efforts individually.

In relation to the scope of the role, a central issue discussed by many participants was the expectation that they needed to represent the views of all service users without any formal mechanisms to consult those views:

…at the end of the day, you are only one voice. You are only one experience… the main issue for me and I've brought up several times is how do we connect with all those other families in the network?

(Participant 11)

In relation to processes and procedures, some participants described a lack of clarity regarding representative recruitment, administrative disorganisation in relation to meetings, a lack of training about the role and ineffective facilitation. Many participants noted that the systems in place were opaque, slow and overly complex:

I cannot understand how complicated they have made this governing process.

(Participant 9)

It's very hard to understand even how it works or what way it should work.

(Participant 2)

One participant described opportunities to shape processes such as advocating for a parent‐led agenda, which was viewed as a positive experience. This enabled family concerns to be raised and prioritised within governance discussions, which they viewed positively:

A parent led agenda that the Governance group agreed to… they presented all of our concerns… we'd get together to make sure that those were in order, they were the priority for the families that we're all representing. So those have been good.

(Participant 8)

Some participants highlighted the absence of mechanisms to incorporate the views of children and young people, particularly those with complex communication needs, and expressed concern that they were often excluded from discussions about services intended to support them. One participant emphasised the need for accessible approaches that would enable all children, including those who were non‐speaking, to contribute to service improvement processes.

The children have, like, I'm talking about, like nonverbal as well. Like, you know, every voice counts. Like, I would love to see something like that set up where it can be directly fed back if they're not at the actual table that they still have a voice, and they're still part of the process.

(Participant 4)

Many participants described developing informal strategies to influence service development when formal mechanisms were ineffective. They described taking on additional responsibilities such as lobbying politicians directly, engaging with the media, and developing their own resources to support the role. These participants felt such actions had a positive impact in getting their voices heard. Some participants also described setting up informal networks to support one another. While this work was experienced as time consuming, it was often viewed as necessary to achieve impact, with one participant noting that:

We went to the media with it. We went to the politicians with it… we got a meeting together… of all the CDNMs [Children's Disability Network Managers], the HSE, the local politicians, both in government and the local councillors and us in the room.

(Participant 7)

For some participants, these efforts were perceived as leading to meaningful change over time. Reflecting on the outcomes of sustained advocacy and lobbying, one participant noted:

So now we're seeing the fruits of that labour.

(Participant 7)

## 6 Theme 3: The Health System as a Controlling and Transactional Organisation

This theme encapsulates participant's conceptualisation of the health system, which they characterised as hierarchical, controlling and transactional. Some participants described communication with members of management as restricted, with many questions left unanswered:

The only person that can talk is the people from the public healthcare… we're limited to their question boxes… and a lot of the time the questions are missed or not answered

(Participant 2)

When meetings were online, some participants shared examples where the chat function was turned off, and they were unable to unmute during meetings. They described this experience as feeling silenced.

All participants described restrictions imposed by organisational members on communication both among representatives and between representatives and the parents they represented. Some participants provided examples where they were unable to share information with other representatives or work collectively to address shared concerns:

The system was afraid of us getting together… they facilitated it with supervision.

(Participant 1)

For some, these practices were described as deliberate efforts to limit the collective voice. As one participant put it:

Nobody talks to each other… they were given out to for talking… it's divide and conquer.

(Participant 6)

Some participants described a lack of transparency within the organisation, particularly in relation to data about services, despite assurances that it would be provided:

I have asked for them so many times, like if you want to be transparent about this, I would like access to data in relation to the number of interventions, the number of children you've seen… and they said, ‘Oh absolutely, we'll get you that’ and then they would never give it to you.

(Participant 9)

Participation was frequently experienced as tokenistic, leaving many participants feeling their contributions were undervalued and not respected. Some participants described being informed about decisions rather than being involved in shaping them. As one participant explained:

Way too many times I would have seen that we were told things as opposed to consulted before decisions were made.

(Participant 10)

Another reflected:

We kind of just felt like we were a ticking box… was any of the information feeding back to the relevant parties who could make the significant changes?

(Participant 11)

In this context, many participants emphasised the importance of improved communication as central to meaningful participation. As one participant noted:

I think that that communication and engagement piece from a national level very much needs to be tackled… I think for me the communication rules all.

(Participant 11)

A lack of understanding by the organisation of what a partnership means was discussed as an issue. Many participants described co‐design not as consultation after decisions had been made, but as a process requiring shared working, open communication, and mutual trust. As one participant noted:

If you're actively involved in a system like that and you want co‐design you want partnership, then you have to communicate absolutely everything, and everyone has to work together.

(Participant 4)

However, some participants described initiatives progressing without representative involvement, reinforcing perceptions that partnership was constrained in practice. Reflecting on the development of a website for the service without representative input, one participant commented:

If co‐design had happened, I don't think it would have got the negative reaction that it did.

(Participant 11)

One participant drew a comparison to another PPIE forum within the health service which they viewed as an authentic partnership. In relation to this PPIE initiative, the participant stated:

I feel the right people are listening there.

(Participant 11)

## 7 Theme 4: Unheard, Unseen, and Unsafe: The Burden of Representation

This theme encompasses the burden of representation as described by the participants. Many participants described a complex interplay of emotional and financial challenges related to the role, which appeared to go unnoticed by health service representatives. At an emotional level, many participants described experiencing stress, anxiety, frustration, exhaustion, and, at times, despair in the role as they were simultaneously navigating services for their own child with complex needs, as well as attempting to represent the needs of other families. For many participants this emotional burden was exacerbated by the fact that they felt their efforts as a parent representative were not having any impact:

Very deflating and disheartening… completely just used and completely lost… putting all our time into this… but actually no progress.

(Participant 4)

In addition to the emotional burden, some participants also described how the logistical demands of organising childcare and the financial costs of their travel and time appeared to go unnoticed by those on senior management salaries:

We are fighting every day for our children… and we're doing this work over and above what anybody else is doing for free… everybody else in the room is on [high salaries] to sit there.

(Participant 1)

Some participants described experiences that made them feel unsafe, particularly when attending national‐level meetings. One participant recalled an incident at a national meeting that they experienced as dismissive and hurtful. The event left them tearful and prompted reflections on the professionalism and conduct of those involved:

…all burst out laughing… I was actually getting tearful because I just couldn't fathom the disrespect… professionally appalling but personally appalling too.

(Participant 6)

Others described more subtle incidences which reinforced feelings of being unseen and unheard:

I always felt like, ‘oh, the little reps there in the corner’… there was a complete lack of appreciation for who we were and what we were able to bring to the table.

(Participant 10)

Some participants described carrying the emotional burden of simultaneously representing other families while navigating their own experiences as parents of children with disabilities. Being a parent of a child with disability heightened their vulnerability, with some describing the experience of simultaneously navigating caregiving and advocacy as existing in a ‘parallel world’. The responsibility of speaking on behalf of families, often without mechanisms for consultation or feedback, created anxiety and self‐doubt. One participant explained:

I worry so much that I'm going to miss something… and I always feel like I could be doing more.

(Participant 5)

Some participants also described the need to be strategic in the role and in knowing how to ‘play the game.’ They stated that playing the game enabled them to continue in the role without fear of impacting negatively their own access to services. Some participants discussed how they learned that conforming to organisational expectations could result in positive outcomes:

If you're willing to say the right things, then magical doors will open up and things will happen that no policy allows.

(Participant 6)

Some participants experienced more positive relationships with health service managers, at a local level. One participant described how these positive relationships had resulted in opportunities to work together effectively:

There was a real…trust there and…level of cooperation… then we sort of developed a relationship with management and just to see how we could work together… opportunities just kept coming

(Participant 7).

Some participants noted that when they were able to create informal mechanisms to connect with other parents, these peer interactions acted as a good source of emotional support:

If there's one good thing about it… it is kind of a support group for parents to meet other parents.

(Participant 9)

## 8 Discussion

This qualitative study explored parents' experiences as representatives within service improvement groups in Ireland's CDNTs. Parents were motivated by a desire to improve services for all families navigating children's disability services. Participants described significant barriers to meaningful participation. These included role ambiguity, limited opportunities to communicate with the wider parent community, restricted access to information, perceived lack of influence on decision‐making processes, and substantial emotional and practical burdens associated with participation. However, findings also revealed examples of positive practice. Some participants described building trusting and productive relationships with local managers, opportunities for collaborative working and the development of informal peer‐support networks with other representatives. Taken together, these findings suggest that while formal structures for family involvement have been established within CDNT governance arrangements, meaningful partnership remains dependent upon contextual factors that support communication, transparency, trust and shared decision‐making.

Participants' accounts pointed to a substantial gap between policy aspirations for partnership and their experiences of participation in practice. Representativeness emerged as a key challenge, with participants expected to represent the views of the wider parent community despite lacking formal mechanisms through which to consult with the families they represented. Concerns regarding representativeness have similarly been identified in international studies of patient and service‐user representatives involved in healthcare working groups and service improvement [9, 10]. These findings resonate with DeCamp et al.'s [24] observation that representative legitimacy depends not merely on holding a representative position but on maintaining active relationships with the communities represented. The present findings extend this literature by demonstrating how the absence of mechanisms for wider engagement can constrain representatives' capacity to fulfil this role. Organisations should therefore strengthen the infrastructure supporting representative participation by enabling regular communication with the wider parent community and providing clear feedback on how representative contributions inform service decisions. This interpretation was reinforced by members of the PPIE parent advisory group, who emphasised that representatives require opportunities to maintain connections with the wider parent community if they are to effectively reflect diverse parent perspectives.

Role ambiguity represented a further challenge to meaningful participation. Participants described uncertainty regarding the scope of the representative role, the expectations placed upon them and the processes through which they could contribute to decision‐making. Similar uncertainty regarding roles and expectations has been identified among patient representatives participating in healthcare working groups [9]. Hult et al. found that representatives' contributions could be hampered by uncertainty surrounding their role and concluded that greater clarity regarding roles and expectations could support more inclusive participation. The present findings illustrate how such ambiguity may lead representatives to interpret and navigate the role themselves, particularly where formal structures and processes provide limited guidance or support.

Participants' experiences also reflect longstanding concerns within the PPIE literature regarding unequal power relations, limited transparency and constrained opportunities for shared decision‐making [4]. Similar challenges have been identified in international empirical research, with representatives describing difficulties in being recognised as equal participants and in speaking up within healthcare teams [7]. Although opportunities for participation existed, many participants struggled to identify tangible evidence of influence and frequently described governance processes as predetermined or resistant to challenge. Viewed through Arnstein's (1969) ladder of participation, these accounts suggest that involvement often remained closer to consultation than full partnership. Participants' experiences also align with Beresford's [25] critique of public participation in health and social care, which highlights how involvement initiatives can be shaped by organisational priorities and existing power structures. From this perspective, the findings suggest a risk that participation may become procedural rather than meaningful. To mitigate this risk, CDNTs should strengthen shared decision‐making processes, increase transparency regarding how decisions are reached and provide clear accountability for how representative input informs service development [25, 26].

The difficulties participants experienced in bringing parent priorities into organisational structures also reflect Beresford's [25] discussion of competing agendas between service users and service providers. Importantly, Beresford [25] emphasises the value of user‐led knowledge and the need to recognise lived experience as legitimate expertise. Although participants brought considerable experiential knowledge and commitment to service improvement, their accounts suggested that this knowledge was not always recognised or valued within formal decision‐making processes. Similar experiences have been reported by representatives in healthcare settings, including difficulties in being recognised and respected as equal members of decision‐making teams [7]. These findings suggest that meaningful participation depends not only on being present within governance structures, but also on whether representatives' experiential knowledge is recognised and able to influence decision‐making. Members of the PPIE parent advisory group similarly highlighted the importance of recognising experiential knowledge as a legitimate form of expertise within service improvement and decision‐making processes.

Although uncommon, participants reported occasions where they were able to shape governance discussions, suggesting that more collaborative forms of participation are achievable within existing structures. One example involved the development of a parent‐led agenda, which enabled family concerns to be collectively identified, prioritised and brought into governance discussions. This aligns with international evidence demonstrating the contribution that representatives can make to healthcare service improvement when they are able to participate actively in improvement processes [10]. The present findings further suggest that providing representatives with opportunities to shape agendas, rather than solely respond to predetermined priorities, may support more meaningful participation and influence.

Alongside issues of recognition and influence, the findings also highlight the considerable burden associated with representative participation, with participants describing significant emotional, practical and financial demands linked to their representative roles. These findings extend emerging literature recognising participation as a form of emotional labour, particularly where contributors experience limited influence or recognition [27]. The accounts presented here suggest that current involvement structures may rely heavily on the goodwill, resilience and persistence of parent representatives. This raises important questions regarding the sustainability, fairness and inclusivity of representative models that depend upon substantial unpaid labour. Participants' accounts also highlighted the value of informal peer support in reducing isolation, suggesting that organisations could facilitate opportunities for representatives to connect with and support one another. More broadly, principles developed within PPIE research emphasise the importance of dedicated support and organisational infrastructure in sustaining involvement [28]. Organisations should therefore consider how representative roles can be adequately supported through payment for time, reimbursement of expenses, administrative support and flexible participation arrangements that recognise parents' existing caregiving responsibilities.

Despite these challenges, participants also described examples of successful advocacy and influence. Engagement with political representatives, service leaders and the media was perceived as increasing the visibility of parent concerns and, in some cases, contributing to meaningful change. Importantly, some of these approaches developed when formal mechanisms for involvement were perceived as ineffective, suggesting that representatives may seek alternative routes to influence where established governance structures do not provide meaningful opportunities for their voices to be heard. To support meaningful representative participation, children's disability services should provide clear role descriptions, structured induction and training, transparent governance processes and accessible communication systems, supported by adequate organisational resources [4, 26].

Despite the challenges identified, some participants described positive relationships characterised by trust, responsiveness, mutual respect and genuine collaboration. These accounts suggested that relationship‐building and cooperation could create further opportunities for representatives and service managers to work together. These experiences echo wider evidence suggesting that successful co‐design is facilitated by strong relationships, open communication and visible evidence that participant contributions lead to change [5, 29]. Similar relational and organisational conditions have been identified in parent co‐design in paediatric healthcare, despite continuing challenges relating to power imbalances, sustaining engagement and demonstrating impact [30]. These positive experiences suggest that meaningful partnership is achievable when representatives are supported by responsive relationships and genuine opportunities for collaboration.

While the study focused on parent representatives, both study participants and members of the PPIE parent advisory group highlighted the importance of ensuring that children's perspectives are also represented within service improvement processes, particularly those of children with complex communication needs. This concern reflects wider evidence that children's participation in service development and governance remains limited despite recognition of their right to have their views heard and influence decision‐making [31, 32]. Emerging evidence suggests that children and young people, including those who are non‐speaking, can contribute meaningfully when accessible and multimodal communication approaches are used [33]. Children's disability services should therefore consider investing in inclusive participation mechanisms that enable children's voices, alongside those of parents, to inform service development.

Taken together, these findings add to existing research on representative involvement in healthcare by showing how unclear roles, difficulties communicating with the wider parent community, limited influence and inconsistent organisational support can restrict meaningful participation. The findings indicate that establishing formal structures for parent representation is not sufficient without the processes and supports needed to enable representatives to participate effectively.

Future research should move beyond documenting barriers alone to examine how and under what circumstances representative participation works well. In particular, realist‐informed research could explore what approaches to representative participation and family involvement work, for whom, in what contexts, and through which underlying mechanisms [34]. Identifying the organisational conditions, leadership approaches and relationship‐building practices that support successful partnership may provide important lessons for strengthening representative participation and family involvement within children's disability services and wider healthcare settings.

## 9 Strengths and Limitations

To our knowledge, this is the first peer‐reviewed qualitative study to explore the experiences of parent representatives participating in Childrens Disability Network Team governance structures in Ireland. The inclusion of participants from across Ireland enabled the exploration of experiences across diverse service contexts and geographical regions, while the qualitative design generated rich, in‐depth accounts of representative participation. A further strength was the involvement of a PPIE parent advisory group throughout the research process, which helped ensure that the study remained grounded in issues of importance to families.

Limitations include the potential for self‐selection bias, recruitment challenges associated with the absence of a centralised register of parent representatives, and the inclusion of parent perspectives only. A further limitation is that information regarding participants' socio‐economic or cultural backgrounds was not collected. Consequently, the potential influence of these factors on experiences of participation and representation could not be explored. As the findings were generated within the Irish CDNT context and reflect experiences at a single point in time, transferability may be limited. Future research incorporating multiple stakeholder perspectives within PPIE and CDNT governance structures, and using longitudinal designs, could provide a more comprehensive understanding of meaningful partnership in children's disability services.

## 10 Conclusions

This study explored the experiences of parent representatives participating in service improvement groups within Ireland's CDNTs. While parents brought considerable commitment, expertise and motivation to the role, participation was constrained by organisational, relational and practical barriers. The findings highlight the importance of strengthening communication pathways, supporting representative legitimacy, reducing the emotional and practical burden associated with participation, and integrating parent and child perspectives into service development.

At the same time, examples of positive partnership identified within the study demonstrate that meaningful collaboration is achievable when participation is supported by transparency, trust and genuine opportunities for influence. Learning from these successful experiences may provide important lessons for strengthening meaningful partnership and family involvement within children's disability services.

## Author Contributions

**Geraldine Moran:** conceptualisation, investigation, writing – original draft, methodology, validation, writing – review and editing, formal analysis, project administration, data curation. **Noreen O'Leary:** conceptualisation, methodology, validation, writing – review and editing, formal analysis, data curation, supervision. **Katie Robinson:** validation, writing – review and editing, methodology, data curation, supervision, conceptualisation. **Hannah Christie:** writing – review and editing, validation, formal analysis, data curation. **Alison Grant:** investigation, validation, methodology, writing – review and editing. **Alyssa Garland:** investigation, validation, methodology, writing – review and editing. **Eileen Doyle:** conceptualisation, methodology, writing – review and editing, validation. **Rosa Tomro‐Hofmann:** conceptualisation, methodology, writing – review and editing; validation. **Aoife Lily Gallagher:** conceptualisation, methodology, investigation, writing – original draft, writing – review and editing, validation, formal analysis, data curation, supervision.

## Funding

The authors have nothing to report.

## Ethics Statement

Ethical approval for this study was granted by the University of Limerick Faculty of Education and Health Sciences Research Ethics Committee (REC Ref: 2025_06_06_EHS).

## Consent

Informed consent was obtained from all participants prior to their participation in the study.

## Conflicts of Interest

The authors declare no conflicts of interest.

## Supporting information


Supporting File 1

Supporting File 2

Supporting File 3

Supporting File 4

## Data Availability

The data sets generated and analysed during the current study are not publicly available due to the need to protect participant privacy and maintain confidentiality. As the data contain potentially identifiable information and participants did not consent to public data sharing, access cannot be provided.

## References

[1] A. Renedo , C. A. Marston , D. Spyridonidis , and J. Barlow , “Patient and Public Involvement in Healthcare Quality Improvement: How Organizations Can Help Patients and Professionals to Collaborate,” Public Management Review 17, no. 1 (2015): 17–34, 10.1080/14719037.2014.881535.

[2] World Health Organization , Framework on Integrated, People‐Centred Health Services: Report by the Secretariat (World Health Organization, 2016), https://apps.who.int/gb/ebwha/pdf_files/WHA69/A69_39-en.pdf.

[3] Healthcare Quality Improvement Partnership , Patient and Public Involvement (PPI) Strategy 2025–2028 (HQIP, 2025), https://www.hqip.org.uk/wp-content/uploads/2025/12/PPI-Strategy-25-28-FINAL-1.pdf.

[4] J. Ocloo and R. Matthews , “From Tokenism to Empowerment: Progressing Patient and Public Involvement in Healthcare Improvement,” BMJ Quality & Safety 25, no. 8 (2016): 626–632, 10.1136/bmjqs-2015-004839. PMC4975844 26993640

[5] Y. Bombard , G. R. Baker , E. Orlando , et al., “Engaging Patients to Improve Quality of Care: A Systematic Review,” Implementation Science 13, no. 1 (2018): 98, 10.1186/s13012-018-0784-z. PMC6060529 30045735

[6] S. Bergholtz , B. Snilstveit , and H. Waddington , “Patient and Public Involvement in Healthcare: A Systematic Mapping Review,” Health Research Policy and Systems 22, no. 1 (2024): 1–15, 10.1186/s12961-024-00789-3.

[7] P. D. Hibbert , Y. Yu , C. J. Molloy , et al., “Perceptions and Experiences of Consumer Representatives on Patient Safety Investigation Teams: A Qualitative Analysis,” Health Expectations 28, no. 3 (2025): e70281, 10.1111/hex.70281. PMC12042694 40304616

[8] N. N. Anderson , K. Dong , G. R. Baker , et al., “Impacts of Patient and Family Engagement in Hospital Planning and Improvement: Qualitative Interviews With Patient/Family Advisors and Hospital Staff,” BMC Health Services Research 22 (2022): 360, 10.1186/s12913-022-07747-3. PMC8932199 35303884

[9] A. Hult , E. Lundgren , and E. Jangland , “Patient Representatives: Crucial Members of Health‐Care Working Groups Facing an Uncertain Role and Conflicting Expectations. A Qualitative Study,” Health Expectations 24, no. 4 (2021): 1197–1206, 10.1111/hex.13249. PMC8369119 33949054

[10] D. Luo , J. McGlashan , K. Lamprell , G. Arnolda , J. Braithwaite , and Y. Zurynski , “Perspectives of Cancer Consumer Representatives on Their Involvement in Healthcare Service Improvement: A Qualitative Study,” BMC Health Services Research 24 (2024): 1324, 10.1186/s12913-024-11669-7. PMC11526679 39482689

[11] Health Service Executive , “Policy Framework for Service Delivery of Children's Disability Network Teams,” 2022a, https://www.cho7cdnt.ie/assets/files/pdf/policy-framework-for-children-s-disability-network-teams.pdf.

[12] Health Service Executive , Setting Up and Sustaining the Family Forum: A Guide for CDNMs and Lead Agencies (Health Service Executive, 2022), https://www.hse.ie/eng/services/list/4/disability/progressing-disability/pds-programme/documents/setting-up-and-sustaining-the-family-forum-a-guide-for-cdnms-and-lead-agencies-2022.pdf.

[13] Health Service Executive , “Roadmap for Service Improvement 2023–2026: Disability Services for Children and Young People,” Health Service Executive, 2023, https://about.hse.ie/publications/roadmap-for-service-imvement-20232026-disability-services-for-children-and-young-people/.

[14] Genio , Family Forum and Family Representative Group Review: Interim Report (Genio, 2024), https://enableireland.ie/sites/default/files/publication/2024.12.3%20Genio%20Interim%20Report%20-%20Family%20Forums%20and%20FRGs%20FINAL%20%281%29.pdf.

[15] L. Allolio‐Näcke , “The Social Constructivist Perspective.” In Cultural Psychology (Springer, 2024), 57–64, 10.1007/978-3-658-45155-4_6.

[16] A. Tong , P. Sainsbury , and J. Craig , “Consolidated Criteria for Reporting Qualitative Research (COREQ): A 32‐Item Checklist for Interviews and Focus Groups,” International Journal for Quality in Health Care 19, no. 6 (2007): 349–357, 10.1093/intqhc/mzm042. 17872937

[17] National Disability Authority , “Review of the Children's Disability Network Team Service Model,” 2026, https://nda.ie/transforming-disability-services/progressing-disability-services-for-children.

[18] S. Staniszewska , J. Brett , I. Simera , et al., “GRIPP2 Reporting Checklists: Tools to Improve Reporting of Patient and Public Involvement in Research,” BMJ 358 (2017): j3453, 10.1136/bmj.j3453. PMC5539518 28768629

[19] K. Malterud , V. D. Siersma , and A. D. Guassora , “Sample Size in Qualitative Interview Studies: Guided by Information Power,” Qualitative Health Research 26, no. 13 (2016): 1753–1760, 10.1177/1049732315617444. 26613970

[20] V. Braun and V. Clarke , Thematic Analysis: A Practical Guide (SAGE, 2021).

[21] V. Braun and V. Clarke , “Reporting Guidelines for Qualitative Research: A Values‐Based Approach,” Qualitative Research in Psychology 22, no. 2 (2025): 399–438, 10.1080/14780887.2024.2382244.

[22] Lumivero , *NVivo (Version 15)* [Computer software] (Lumivero, 2023), https://lumivero.com/products/nvivo/.

[23] V. Braun and V. Clarke , “Reflecting on Reflexive Thematic Analysis,” Qualitative Research in Sport, Exercise and Health 11, no. 4 (2019): 589–597.

[24] M. DeCamp , S. E. Brewer , and V. Dukhanin , “Patient, Public, Consumer, and Community Engagement: From Consucrat to Representative. Comment on ‘The Rise of the Consucrat’,” International Journal of Health Policy and Management 10, no. 8 (2020): 503–506, 10.34172/ijhpm.2020.148. PMC9056203 32823377

[25] P. Beresford , “Public Participation in Health and Social Care: Exploring the Co‐Production of Knowledge,” Frontiers in Sociology 3 (2019): Article 41, 10.3389/fsoc.2018.00041. PMC8022568 33869395

[26] J. Ocloo , S. Garfield , B. D. Franklin , and S. Dawson , “Exploring the Theory, Barriers and Enablers for Patient and Public Involvement Across Health, Social Care and Patient Safety: A Systematic Review of Reviews,” Health Research Policy and Systems 19, no. 8 (2021): 8, 10.1186/s12961-020-00644-3. PMC7816359 33472647

[27] K. Liabo , L. Asare , P. Ruthen , J. Burton , P. Staunton , and J. Day , “Emotion in Public Involvement: A Conceptual Review,” Health Expectations 27, no. 3 (2024): e14020, 10.1111/hex.14020. PMC10951420 38504467

[28] C. A. Chew‐Graham , “Sustaining Patient and Public Involvement and Engagement in Research,” Health Expectations 21, no. 6 (2018): 937–938, 10.1111/hex.12848. PMC6250856 30467954

[29] C. Clarke , A. Kouroupa , R. Royston , et al., “We're All in This Together”: Patient and Public Involvement and Engagement in Developing a New Psychosocial Intervention for Adults With an Intellectual Disability Who Display Aggressive Challenging Behaviour,” Research Involvement and Engagement 11, no. 1 (2025): 20, 10.1186/s40900-025-00675-6. PMC11887246 40050986

[30] J. Suna , D. Tanti , G. Merlo , P. Bryant , B. McMullan , and L. Hall , “Evaluating Co‐Design Approaches With Parents in Paediatric Healthcare: A Systematic Review,” BMC Health Services Research 25, no. 1 (2025): 1416, 10.1186/s12913-025-13495-x. PMC12570435 41152904

[31] G. Gurung , A. Richardson , E. Wyeth , L. Edmonds , and S. Derrett , “Child/Youth, Family and Public Engagement in Paediatric Services in High‐Income Countries: A Systematic Scoping Review,” Health Expectations 23, no. 2 (2020): 261–273, 10.1111/hex.13017. PMC7104655 31981295

[32] L. Lundy , “‘Voice’ Is Not Enough: Conceptualising Article 12 of the United Nations Convention on the Rights of the Child,” British Educational Research Journal 33, no. 6 (2007): 927–942, 10.1080/01411920701657033.

[33] R. Lyons , C. Carroll , A. Gallagher , R. Merrick , and H. Tancredi , “Understanding the Perspectives of Children and Young People With Speech, Language and Communication Needs: How Qualitative Research Can Inform Practice,” International Journal of Speech‐Language Pathology 24, no. 5 (2022): 547–557, 10.1080/17549507.2022.2038669. 35188849

[34] R. Pawson and N. Tilley , Realistic evaluation (Sage, 1997).

