# Supplementary material for: Parent Representatives' Experiences of Participating in Children's Disability Network Team Service Improvement Groups in Ireland: A Qualitative Study
Source: Health Expect. 2026 Nov 5;29(6):e70962. doi: 10.1111/hex.70962 (PMC13735219; doi:10.1111/hex.70962)
Supplement: Supplementary file 2 [file HEX-29-e70962-s002.docx]

### INTERVIEW TOPIC GUIDE

**Study title:**

**Experiences of parent representatives on Health Service Executive Children's Disability Network Team service improvement groups in Ireland**

1. Tell me why you got involved as a parent representative.
2. Tell me what was going on for you or what stage your child was at when you got involved in these groups.
3. Tell me about your experience of the process.
4. What do you think was/is missing from the process?
5. Would you get involved again as a parent rep? Yes/No Why? Why not?
6. If you could provide three recommendations to improve the process, what would they be?
7. Is there anything else you would like to add that you haven’t had a chance to say?
