# Supplementary material for: Parent Representatives' Experiences of Participating in Children's Disability Network Team Service Improvement Groups in Ireland: A Qualitative Study
Source: Health Expect. 2026 Nov 5;29(6):e70962. doi: 10.1111/hex.70962 (PMC13735219; doi:10.1111/hex.70962)
Supplement: Supplementary file 3 [file HEX-29-e70962-s003.docx]

**Appendix 4:**

**Analytic progression; Open Codes	Categories	Themes**

| **Themes** | **Category Examples** | **Open Code Examples** |
| --- | --- | --- |
| **Theme 1: From the individual to the collective, parent motivations to become a parent representative** | Personal experiences of unmet need and service failure  Aspirations for advocacy and change | PDS/CDNT systems failure.  Trying to get supports.  Frustration with services.  Doing it for their child.  Being a voice.  Helping others.  Influencing services.  Improving experiences for families |
| **Theme 2: An ambiguous and informal role, with unintended consequences** | Unclear role expectations and representation responsibilities  Inadequate structures and processes for meaningful participation | Unclear representative role.  Lack of role definition.  Confusion about expectations.  Difficulty connecting with wider parent networks.  Administrative disorganisation. Lack of training. Limited influence within formal structures. Exclusion of children's voices |
| **Theme 3: The health system as a controlling and transactional organisation** | Organisational control of communication and participation  Tokenistic involvement and lack of transparency | Control over information.  Restricted communication with management. Questions ignored or unanswered. Muted participation in meetings  Lack of access to service data.  Unfulfilled promises of transparency.  Decisions made without consultation.  Feeling like a tick-box exercise |
| **Theme 4: Unheard, unseen, and unsafe: The burden of representation** | Emotional burden of representation  Practical and financial burden of participation | Frustration.  Despair.  No visible impact from efforts.  Unpaid labour.  Financial burden.  Sacrifice of personal time |
