# Supplementary material for: Parent Representatives' Experiences of Participating in Children's Disability Network Team Service Improvement Groups in Ireland: A Qualitative Study
Source: Health Expect. 2026 Nov 5;29(6):e70962. doi: 10.1111/hex.70962 (PMC13735219; doi:10.1111/hex.70962)
Supplement: Supplementary file 4 [file HEX-29-e70962-s004.docx]

| **Appendix 2: GRIPP2-SF** (Staniszewska et al., 2017) | |
| --- | --- |
|  | **Description in Current Study** |
| **1: Aim** | PPIE was undertaken to ensure that the study reflected the priorities, experiences, and perspectives of parents of children accessing Children's Disability Network Team (CDNT) services and to enhance the relevance and credibility of the research. |
| **2: Methods** | PPIE contributors were involved throughout the study. Three parents with relevant lived and representative experience contributed to the research. Two parents had lived experience of Children's Disability Network Team (CDNT) services, while the third was a parent of an adult with complex needs and had substantial experience of PPIE and representative forums. Together, they contributed to the study design, development of the semi-structured interview guide, recruitment approaches, dissemination through parent networks, review of emerging interpretations, and refinement and naming of themes. During the analysis and write-up phase, one parent contributor with lived experience of CDNT services participated in a 90-minute consultation meeting with the research team. Two contributors subsequently reviewed the completed manuscript. |
| **3: Study results** | PPIE contributors improved the relevance and accessibility of interview questions, informed recruitment strategies, contributed lived-experience perspectives during analysis, advised on theme titles, and identified priority issues for inclusion in the discussion. No negative outcomes were identified. |
| **4: Discussion and conclusions** | PPIE influenced the design, recruitment, interpretation, reporting, and presentation of findings. Contributor feedback informed theme refinement and the discussion section, helping to ensure that findings resonated with parent experiences. No negative effects of PPI involvement were identified. |
| **5: Reflections/critical perspective** | Sustained involvement of parent contributors throughout the research process strengthened the relevance and credibility of the study. The interview topic guide was developed collaboratively with the PPIE parent advisory group. The research team recognised that some areas prioritised by the group reflected concerns arising from their own experiences of representation. Reflexive discussion of this influence informed the interview process, and interviewers used balancing probes to explore both positive and negative experiences of participation. |
